# Alpha-Synuclein and Lipids: The Elephant in the Room?

**DOI:** 10.3390/cells10092452

**Published:** 2021-09-17

**Authors:** Alessia Sarchione, Antoine Marchand, Jean-Marc Taymans, Marie-Christine Chartier-Harlin

**Affiliations:** Univ. Lille, Inserm, CHU Lille, UMR-S 1172—LilNCog—Lille Neuroscience and Cognition, F-59000 Lille, France; Alessia.Sarchione@inserm.fr (A.S.); Antoine.Marchand@inserm.fr (A.M.); jean-marc.taymans@inserm.fr (J.-M.T.)

**Keywords:** α-synuclein, exocytosis, genetics, lipids, membranes, Parkinson disease, SNARE complex, synapse, vesicle fusion, therapeutic target

## Abstract

Since the initial identification of alpha-synuclein (α-syn) at the synapse, numerous studies demonstrated that α-syn is a key player in the etiology of Parkinson’s disease (PD) and other synucleinopathies. Recent advances underline interactions between α-syn and lipids that also participate in α-syn misfolding and aggregation. In addition, increasing evidence demonstrates that α-syn plays a major role in different steps of synaptic exocytosis. Thus, we reviewed literature showing (1) the interplay among α-syn, lipids, and lipid membranes; (2) advances of α-syn synaptic functions in exocytosis. These data underscore a fundamental role of α-syn/lipid interplay that also contributes to synaptic defects in PD. The importance of lipids in PD is further highlighted by data showing the impact of α-syn on lipid metabolism, modulation of α-syn levels by lipids, as well as the identification of genetic determinants involved in lipid homeostasis associated with α-syn pathologies. While questions still remain, these recent developments open the way to new therapeutic strategies for PD and related disorders including some based on modulating synaptic functions.

## 1. Introduction

Parkinson’s disease (PD) is one of the main neurodegenerative disorders, whose development is mainly due to the combined result of environmental factors and genetic predispositions, and based on the age at which symptoms appear, can be classified as juvenile, early onset, or late onset [1]. The neurodegeneration mainly affects the survival of dopamine producing neurons of the substantia nigra pars compacta, and both the premature degeneration of dopaminergic neurons and accumulation of protein-rich aggregates, called Lewy bodies, are the main neuropathological hallmarks of PD [2]. Post-mortem diagnosis of pre-symptomatic stages of the disease is based on the identification of these inclusion bodies, which develop as spindle-like Lewy neurites in cellular processes and as globular Lewy bodies in neuronal cell bodies [3]. These hallmarks are associated with consistent activation of microglia surrounding degenerating dopaminergic neurons in the substantia nigra, suggesting an important role of the immune system in this disorder [4]. At present, no curative treatments for PD are available, putting forward the need to better understand the mechanisms leading to the neurodegeneration of the nigrostriatal system. This might come from a better understanding of the role of a key protein involved in this disorder, namely alpha-synuclein (α-syn).

**Figure 1:**
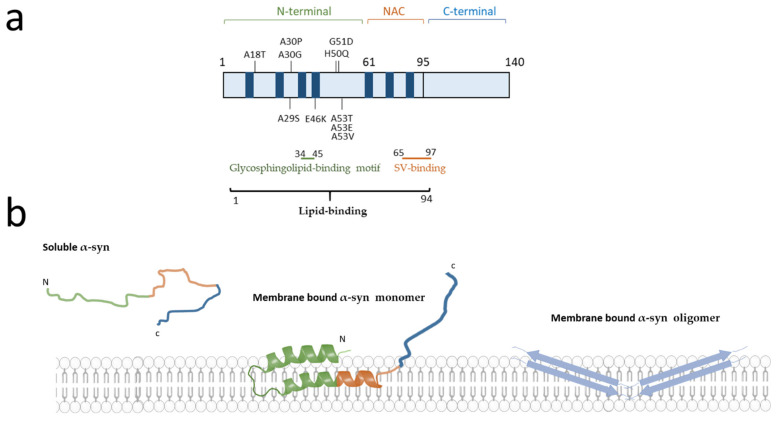
Schematic representation of α-synuclein (α-syn) mutations and lipid binding regions. (**a**) Schematic representation of the domain structure of α-syn. The α-syn is composed of three domains: the N-terminal domain (green), the NAC domain (orange) and the C-terminal domain (blue). Four confirmed pathogenic autosomal dominant missense mutations (A30P, G51D, A53E, A53T) as well as six putatively pathogenic mutations (A18T, A29S, A30G, E46K, H50Q, A53V) are depicted [11]. In blue are represented the seven KTKEGV hexameric repeats spanning from the N-terminus to the non-amyloid β-component (NAC) domain. The lipid binding regions are represented by lines of different colours (black = lipid binding, green = glycosphingolipid-binding motif and red = synaptic vesicles (SV)). (**b**) Schematic representation of the different conformations of the α-syn. α-syn is present in the cytosol as unfolded monomer. Binding of α-syn to lipids induces a conformational change of α-syn N-terminal region, which acquires an α-helix secondary structure. The oligomers penetrate into the lipid bilayer with a β-sheet structure. The membrane image is adapted from Servier Medical Art (smart.servier.com, accessed on 19 July 2020) licensed under a Creative Commons Attribution 3.0 Unported License.

The α-syn protein is encoded by the *Non-A4 Component Of Amyloid Precursor* (*SNCA*) gene that is located at the PARK1/4 locus on chromosome 4q21 and consists of six protein coding exons [5,6,7]. While PD is mainly sporadic, several deleterious or potentially deleterious mutations in this gene (A18T, A29S, A30G, A30P, E46K, H50Q, G51D, A53E, A53T, and A53V) have been linked to familial parkinsonism [8,9,10,11] (Figure 1a). Further evidence, including triplication [12] and duplication of the *SNCA* gene locus [13,14], demonstrates that the sole overexpression of α-syn can lead to the disease. Families with *SNCA* mutations or locus multiplications are relatively rare; however, several case control studies and genome-wide association studies (GWAS) demonstrated that polymorphisms at this gene locus also are moderate risk factors for PD [15,16,17]. Furthermore, post-transcriptional effects on *SNCA* transcripts, such as usage of alternative start sites and variable UTR lengths exist [18,19], leading to more than 40 transcripts, at least some of which are associated to PD [20]. Epigenetic deregulation in the *SNCA* gene is also associated with idiopathic PD [21]. In addition, *SNCA* copy number variant mosaicism has been reported [22,23,24]. Further studies are needed to confirm the roles of transcript, epigenetic, and mosaicism variants in the pathogenesis of PD. Overall, the *SNCA* gene is one of the most important genetic determinants involved in the pathogenesis of PD [25,26].

Additional arguments point to the major role of α-syn in neurodegenerative disorders. Indeed, it has long been established that aggregated α-syn is a hallmark of synucleinopathies, including the presence of α-syn positive Lewy bodies in the neurons of PD, dementia with Lewy bodies [27] and some variants of Alzheimer’s disease [27,28]. In addition, aggregated α-syn has been observed in glial cells in multiple system atrophy [29]. Spontaneous conversion of soluble unfolded α-syn monomers into aggregates leads to accumulation of α-syn in neurons. The most common form of α-syn is thought to be monomeric and found in the cytoplasm of neuronal cell models [30], whereas under pathological conditions α-syn is thought to form oligomers (Figure 1b). Intriguingly, under physiological conditions, α-syn is able to form helically folded tetramers that might be more resistant to aggregation. However, these data need still to be better understood [31]. Conversely, the spread of insoluble α-syn propagation from cell-to-cell is currently considered as a mechanism to explain the pathological progression of disease along synaptically connected regions of the brain [32,33]. Furthermore, many studies in post-mortem brains, indicate that the degree of microglial activation in PD is directly correlated with α-syn deposition, suggesting that α-syn may be directly involved in activating the innate immune system [4]. Similarly, recent data have shown overexpression of α-syn in human induced pluripotent stem cells (iPSC) derived neurons and in neuronal tissues of non-human primates after viral infection, further bolstering the hypothesised link between immune system challenge and synucleinopathies [34].

In addition, the α-syn protein is involved in a wide range of processes impaired in PD pathophysiology including transport of synaptic vesicles (SV), regulation of dopamine release, and vesicular trafficking. Indeed, α-syn physiologically interacts with membrane lipids (Figure 1b) and proteins in order to regulate synaptic plasticity and neurotransmitter release [35]. A current hypothesis is that α-syn dysfunction can lead to defects in vesicular trafficking and several studies conducted in worm, yeast, fly, and mouse models tend to confirm this assumption [36]. Further evidence supporting the ability of α-syn to regulate membrane trafficking processes is directly correlated with its interaction with membrane lipids and several proteins, especially at the synapse. Among the partners of α-syn, a crucial role has emerged for instance for SNARE proteins (soluble N-ethylmaleimide-sensitive factor (NSF) attachment protein (SNAP) receptors), which represent the core machinery mediating vesicle trafficking and membrane fusion. The orchestrated coordination of α-syn and SNARE proteins allows the regulation of synaptic plasticity and neurotransmitter release [35,37]. Interestingly, an emerging dimension to the role of α-syn in membrane trafficking is the importance of membrane lipid composition, with recent evidence showing for example that membrane lipid composition modulates the role of α-syn in neurotransmitter release [38]. Thus, we aim to examine in a first step the physical relationship between α-syn and lipids in the context of plasma and SV membranes. Secondly, we will describe the implications of these interactions on synaptic functions of α-syn, including docking, exocytosis, and recycling of SV. The final goal is to discuss the lipid deregulations in PD and potential therapeutic strategies for synucleinopathies.

## 2. α-Synuclein and Its Relationship with Lipid Membranes

α-syn was originally described as a protein enriched at the synapse [39] and was later identified as a component of Lewy bodies in PD [40]. Of particular importance recently, we learned that these inclusions are also enriched in lipid membranes and degenerated organelles [41]. These data first suggested a role for α-syn at the synapse and recent advances on the composition of Lewy bodies highlight a strong relationship between α-syn and membranes as well as lipids. Moreover, the role of α-syn in synaptic activity implies the need to decipher the mechanism of interaction of α-syn with biological membranes.

### 2.1. α-Synuclein Structure and Interaction with Lipids

Biophysical studies reveal that α-syn interacts with lipid components of biological membranes in different manners. The specific nature, affinity, and functional effects of these interactions have been extensively investigated by in vitro studies performed on artificial membrane systems of different levels of complexity (summarised in Table 1).

The studies in membrane-mimicking models investigate the interaction between different classes of lipids and the three α-syn domains: the positively charged N-terminal domain (residues 1–60), a central hydrophobic NAC (non-amyloid β-component) domain (residues 61–95), and the acidic C-terminal tail (residues 96–140). The different domains and motifs of α-syn are schematically depicted in Figure 1. The basic character of the N-terminal domain allows the formation of electrostatic interactions with acidic negatively charged membrane lipids [45] particularly enriched in the membrane of SV [46]. The N-terminal domain shows an affinity for glycosphingolipids and, specifically, the residues 34–45 have been proposed as a cell surface lipid-binding motif bearing a solvent-accessible aromatic residue [47]. It should be noted that such a domain is also present on other proteins responsible for neurodegeneration such as prion protein and amyloid β [48]. The binding of the N-terminal domain of α-syn to lipids induces a conformational change from a random-coil to a more stable α-helix structure [49]. 

The α-syn protein sequence has several characteristic imperfect repeats of 11 amino acids extending from the N-terminus to the NAC domain with a highly conserved hexameric sequence (KTKEGV), which is also present in the α-helix motif of the lipid-binding domains of apolipoproteins A2 [42]. These repeats have the propensity to adopt an α-helical structure upon binding with negatively charged phospholipid membranes. Studies on sodium dodecyl sulphate-micelles suggest that α-syn-micelle bonds involve a long α-helical region (from residue 1–94) interrupted by a short linker including residues 42, 43 and 44. These data are in contrast to previous evidence from Davidson et al. showing the existence of five α-helices of α-syn bound to liposomes [50]. The two models are not considered mutually exclusive and the switch between the two conformations depends on membrane lipid rearrangement and organisation [51]. The central NAC domain is the most hydrophobic part of α-syn and is prone to acquire a β-sheet conformation [52]. It represents the domain leading to the nucleation of α-syn in oligomer formation. The NAC region might be partially inserted into the lipid bilayer [53], but its most important role is to act as a modulator of α-syn affinity for lipid membranes [54]. 

The C-terminal domain, enriched in proline residues, is an unstructured region likely due to its low hydrophobicity and confers flexibility to the protein. The C-terminus is weakly associated with the membrane [54], but it has recently been shown that calcium increases the membrane association of this domain. The random coil configuration of the acidic carboxylic tail is conserved also in the α-syn lipid bound state [55]. In addition, this α-syn domain undergoes several post-translational modifications, the best known being the S129 phosphorylation that accumulates within Lewy bodies [56].

### 2.2. α-Synuclein and Lipid Bilayers

Biological membranes exhibit a heterogeneity in lipid composition as well as asymmetry in the proportions and distribution of lipids between the two leaflets of the lipid bilayer. This asymmetric lipid composition will influence the binding affinity of α-syn to the presynaptic and SV membranes.

#### 2.2.1. Presynaptic Membrane Composition and α-Synuclein Binding Affinity

**Figure 2:**
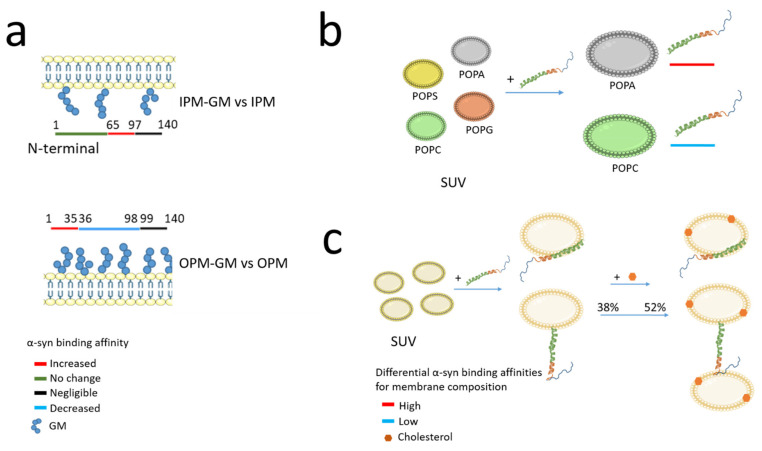
Schematic representation of differential affinity of α-synuclein (α-syn) for the inner or outer plasma membrane (IPM or OPM) as well as for vesicles according to their lipid compositions. (**a**) Differential affinity of α-syn for IPM and OPM according to differences in the amount of gangliosides (GM): IPM-GM versus (vs) IPM or OPM-GM versus OPM as described by Man et al. (2021) [38]. (**b**) Differential affinity of α-syn for artificial vesicles based on their membrane composition. α-syn has a 60 times higher affinity for 1-palmitoyl-2-oleoyl-*sn*-glycero-3-phosphate (POPA) than 1-palmitoyl-2-oleoyl-phosphatidyl-l-serine (POPS) and 1-palmitoyl-2-oleoyl-*sn*-glycero-3-phosphoglycerol (POPG) and very low affinity for 1-palmitoyl-2-oleoyl-sn-glycero-3-phosphocholine (POPC). (**c**) Effect of cholesterol on the conformation of α-syn. α-syn interacts with vesicles to promote fusion between 2 vesicles as described by Fusco et al. (2016) [61] and Man et al. (2020) [62]. Upon interaction with small unilamellar vesicles (SUV) composed of 1,2-dioleoyl-sn-glycero-3-phospho-ethanolamine (DOPE), 1,2-dioleoyl-sn-glycero-3-phospho-L-serine (DOPS), 1,2-dioleoyl-sn-glycero-3-phosphocholine (DOPC), α-syn exists in multiple different conformational states. These include the α-helical state covering the 1–97 region (top) and a conformational state interacting with the membrane through N-terminal residues 1–25. It has been proposed by Man et al. (2020) that the presence of cholesterol in the SUV composition induces an increase in the proportion of α-syn with the conformational state described at the bottom from 38% to 52%, leading to the 65–97 region being available to interact with a second SUV [62]. This suggests that cholesterol promotes the docking of the vesicles-mediated by α-syn.

Biological membranes are mainly composed of three different types of lipids classified as phospholipids, glycolipids, and cholesterol [57]. Studies on the lipid composition of the plasma membrane (PM) reveal that, among the phospholipids, the most represented in membrane include phosphatidylcholine (PC), phosphatidylethanolamines (PE), sphingomyelin, and cholesterol. These classes of lipids are found in both leaflets of the membrane. Nevertheless, biochemical analyses revealed the asymmetric distribution of lipids between the two leaflets of the bilayer called inner PM (IPM on the cytosolic side) and outer PM (OPM on the extracellular side). Interestingly, under physiological conditions, phospho-L-serine (PS), phosphatidylinositol (PI) and phosphatidylinositol phosphates (PIPs) are more specifically present on the IPM. In contrast, gangliosides (GM) and cerebrosides are more specific to OPM [58]. Importantly, based on this differential distribution, the relationship of α-syn to the two leaflets was studied in a series of in vitro experiments by Man et al. using artificial membranes as models reflecting the same asymmetric distribution between the two leaflets of biological membranes [38]. The authors show that the binding of α-syn to either leaflet of the PM is quite different with α-syn having a strong affinity for IPM compared to OPM with the N-terminal region having the higher binding strength. This study supports the hypothesis of double-anchor mechanism whereby α-syn binds simultaneously to the IPM through its N-terminal region and to SV through a motif located in the NAC domain (residues 65–97) which has a weak affinity for IPM. Moreover, knowing that many classes of lipids are altered in neurodegenerative disorders (Table 2), Man et al. then investigated the α-syn binding affinity with IPM or OPM according to the enrichment or not of GM components (Figure 2a). Indeed, GM has emerged as an important factor in maintaining neuronal functions [59] and, moreover, GM concentration is altered in neurodegenerations with 22% reduction in brain GM content in men with PD, no differences in women with PD [60] and a 45% reduction in GM content observed in late stages of Alzheimer’s disease. Assessment of the affinity of α-syn for OPM and IPM according to GM enrichment in both leaflets draws further attention to the role of GM on α-syn binding region. A six-fold increase interaction of the α-syn region 65–97 was observed in IPM-GM compared to IPM, while the N-terminal region kept the same strong affinity of binding for IPM-GM as for IPM. These results were confirmed also by the conformation analyses using chemical exchange saturation transfer experiments [38]. Similarly, α-syn shows stronger binding to OPM-GM than to OPM. In particular, the residues 1–35 of α-syn at the N-terminus show the higher affinity to OPM-GM, whereas both regions 36–98 and the C-terminal region 99–140 have low affinity or no binding, respectively [38].

These observations support the ability of α-syn to drive the docking of synaptic-like small unilamellar vesicles (SUV) to IPM in a concentration-dependent manner. Furthermore, if cholesterol levels are disturbed in PD patients, α-syn binding to OPM showing increased GM could be favoured. Therefore, the differential binding of α-syn to the two leaflets of the bilayer may have important implications in the synaptic activity of α-syn as described later in Section 3.1.

#### 2.2.2. Lipid Rafts and α-Synuclein Interaction

On the PM, there are lipid microdomains called lipids rafts characterised by combinations of glycosphingolipids, cholesterol, and receptor proteins. Other lipids, such as relatively saturated phospholipids. have often been associated with raft-like environments [95]. They form functional platforms involved in the regulation of cellular functions and are present in both the inner and outer leaflets. The interaction of α-syn with lipid rafts is crucial in ensuring the synaptic localisation of α-syn. Indeed, knowing that in OPM glycosphingolipids are mostly present in sphingomyelin and cholesterol enriched lipid rafts, Fantini et al. determined the following ranking for the interaction of α-syn with glycosphingolipids [47]: GM3 > Gb3 > GalCer-NFA > GM1 > sulfatide > GalCer-HFA > LacCer > GM4 > GM2 > asialo-GM1 > GD3. Interestingly, the presence of GM3 stimulated the insertion of α-syn into sphingomyelin containing monolayers and promoted the integration of α-syn in raft-like membrane domains [47]. Furthermore, this association of α-syn with lipid rafts is dependent on ergosterol content and can be abrogated by depletion of cholesterol or by the presence of the α-syn A30P mutation. These two parameters also modify the preferential localisation of α-syn towards detergent-resistant fractions, corresponding to lipid raft domain of yeast membranes [96]. Note that Fortin et al. demonstrated in cellulo that synaptic localisation depends strongly on its interaction with the lipid rafts. Indeed, changes in lipid raft composition or affinity of α-syn in their binding may compromise the α-syn localisation and consequentially its normal function at the synapse in mouse brain [97]. Interestingly, Perissinotto et al. proposed another mechanism of preferential interaction, in which heavy metals play an important role in defining the lipid raft localisation of α-syn species [98]. In this study using atomic force microscopy, a thinning of the PM in the absence of ferrous cations Fe^2+^ and in the presence of monomers is observed. Knowing that heavy metal ions contribute to aggregations of monomers, the authors exposed the bilayer membrane model to Fe^2+^ and observed oligomer-like structures as expected. Interestingly, these aggregates were preferentially directed towards the lipid raft phase of the bilayer model [98]. In parallel, the authors show that the A53T mutated α-syn exhibited a greater and faster membrane interaction compared to wild type (WT) α-syn. If such models also exist in pathological conditions, this would further strengthen the role of lipids in PD pathophysiology.

### 2.3. α-Synuclein and Synaptic Vesicles

A large number of biophysical studies on α-syn and lipid interactions aimed to define the specificity and affinity of α-syn for synaptic-like vesicles as a function not only of lipid composition, but also of other parameters, including the size and curvature of vesicles. 

#### 2.3.1. α-Synuclein and Membrane Curvature

α-syn is capable of generating membrane curvature [99,100] and the synaptic concentration of α-syn is sufficient to induce membrane bending [101]. The curvature process occurs through the insertion of N-terminal region of α-syn into the membrane in a manner similar to other amphipathic helical proteins, such as endophilin [101]. Indeed, α-syn belongs to the class of proteins that can initiate a wedge in the bilayer (the amphipathic helices (9–41 AA)) and binds preferentially to pre-curved bilayers, where curvature has created a gap in lipid packing. Such a protein is considered as a curvature generator and curvature sensor [102]. Thanks to this ability, α-syn as well as other proteins such as β-syn and apolipoprotein A-1 are able to convert large vesicles into highly curved membrane tubules and vesicles [99]. However, compared to other curvature sensor proteins, α-syn does not use a bin/amphiphysin/rvs (BAR) domain and, therefore, has a lesser ability to induce tubulation compared to other proteins such as endophilin A1. 

When studying the effect of different forms of α-syn, only monomeric, but not tetrameric, α-syn is able to induce membrane curvature. Moreover, the A30P mutant of α-syn, characterised by a distortion in its N-terminal domain and consequent disruption of α-helix formation, has a weak membrane binding, thus losing the ability to drive the membrane curvature [101]. In addition, the alterations in membrane trafficking observed in PD models of α-syn overexpression [103] were potentially associated with alterations in membrane curvature and membrane disruption induced by overexpression of α-syn [99]. Thereby, the membrane curvature mediated by α-syn may represent a crucial process allowing α-syn to fulfil a functional role in vesicle trafficking and vesicle exocytosis.

#### 2.3.2. α-Synuclein Affinity According to Vesicle Composition

The lipid composition of vesicles deeply affects the binding, the state, and the solubility of α-syn, as documented above. Although physical interaction with lipid components of vesicles is crucial in the synaptic activity of α-syn, the affinity of α-syn for vesicles can change depending on the vesicle composition, size, and lipid packaging (Figure 2).

The α-syn shows a higher affinity for synaptic-like vesicles composed of negatively charged phospholipids, particularly phosphatidyl-glycerol and PS [104,105]. Of note, other components such as PC, PE, and PI, as well as cholesterol, sphingomyelin, and hexosylceramide are part of the SV membranes [106]. Moreover, an in vitro study performed on vesicles composed of anionic lipids 1-palmitoyl-2-oleoyl-sn-glycero-3-phosphoserine (POPS), 1-palmitoyl-2-oleoyl-*sn*-glycero-3-phosphoglycerol (POPG), or 1-palmitoyl-2-oleoyl-*sn*-glycero-3-phosphate (POPA) in 1:1 mixed with the zwitterionic 1-palmitoyl-2-oleoyl-*sn*-glycero-3-phosphocholine (POPC) shows that α-syn preferentially binds POPA with a 60-times higher affinity than POPS and POPG and very low affinity for POPC, confirming the importance of negatively charged lipid in α-syn binding (Figure 2b) [107].

α-syn binds preferentially to SUV rather than large unilamellar vesicles (LUV) of the same composition, most likely due to differences in phospholipid packing on the vesicle surface [107]. α-syn also shows an intrinsic affinity for highly curved lipid surfaces, which can be modulated by specific lipid components and the presence of bilayer defect. Other properties of the lipid bilayer could affect the α-syn binding including charge and surface hydrophobicity [108]. The interaction of α-syn with SUV composed of DOPE, DOPS, and DOPC favours the conformation of α-syn with the N-terminal region attached to the SUV and the region 65–97 available to bind another vesicles (Figure 2c) [62].

In addition, α-syn post-translational modifications could deeply affect the lipid interactions. For instance, α-syn acetylation increases the lipid-binding affinity [109] and specifically the acetylation of N-terminal α-syn is able to enhance binding to PC micelles and SUV with high curvature (16–20 nm) [110]. Phosphorylation of residue S129 increased or reduced the lipid-binding affinity of A30P and A53T, respectively [111]. Moreover, α-syn phosphorylation at residue Tyr39 could affect the α-syn conformation and, thus, the ability to bind lipids [112].

## 3. α-Synuclein Function in Exocytosis

The presynaptic localisation and the association of α-syn with lipids and the co-localisation of α-syn with proteins involved in exocytosis, such as Rab protein family members and soluble N-ethylmaleimide-sensitive factor attachment protein receptors (SNAREs), support the involvement of α-syn in synaptic plasticity and synaptic vesicle regulation [35]. Trafficking of SV is a process characterised by different steps including formation of the vesicles, tethering, docking, and fusion [113]. SV cluster at the presynaptic membrane and are then released by exocytosis, enabling communication between neurons.

**Figure 3:**
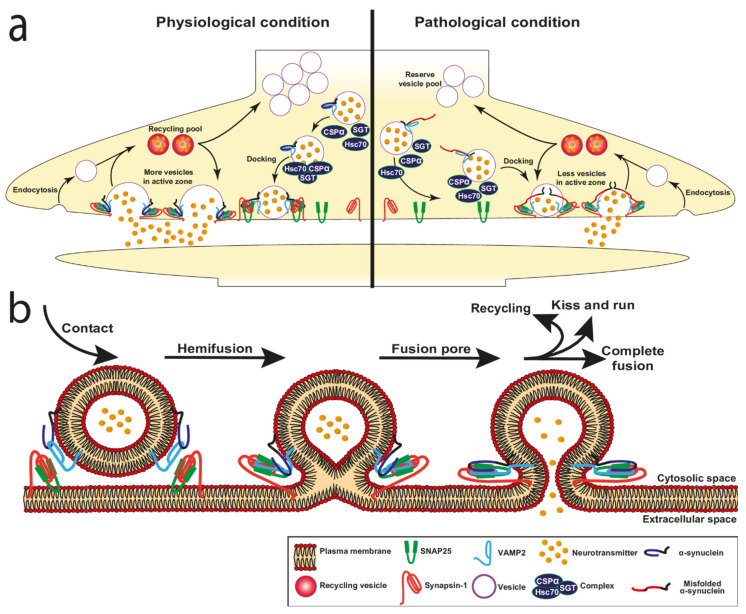
Schematic hypothesis of the role of α-synuclein (α-syn) in exocytosis. (**a**) α-syn, under physiological condition (**left** panel), interacts with the soluble N-ethylmaleimide-sensitive-factor attachment protein receptor (SNARE) vesicle-associated membrane protein 2 (VAMP2) on the synaptic vesicles (SV) surface, drives the docking of the SV to the active zone and regulates the formation of the tripartite SNARE-complex. Others synaptic partners including synapsin-1 and complexin act in the complex stabilisation. The SNARE-complex regulates the fusion of the SV with the synaptic membrane. After cargo release, the vesicles are recycled. Under pathological condition (**right** panel) aberrant forms of α-syn have a stronger binding affinity for VAMP2. The reduced availability of unbound VAMP2 molecules inhibits the SNARE complex formation and reduces the number of vesicles in the active zone. (**b**) α-syn actively participates in exocytosis by regulating SNARE complex formation and vesicle fusion events. Indeed, α-syn favours dilatation/closure of the fusion pore as well as regulates the kiss and run exocytosis. SNAP25 = synaptosome associated protein 25, CSPα = cysteine-string protein-α, Hsc70 = heat shock cognate 70, SGT = small glutamine-rich tetratricopeptide repeat-containing protein α.

**Figure 4:**
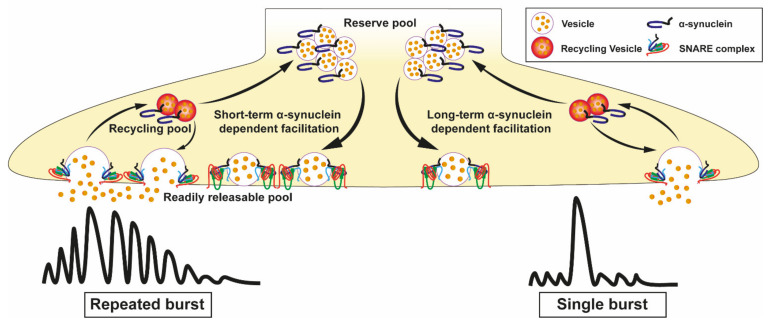
Exocytosis events mediated by α-synuclein (α-syn) are influenced by action potential bursts. α-syn influences exocytosis in different ways depending on the duration of action potential bursts. It is known that high frequency stimulation is responsible for the exhaustion of dopamine storage pool [158]. Dopamine release is promoted by α-syn when action potential bursts are separated by short intervals or reduces release when the interval between consecutive bursts is in the range of minutes [156].

It has been demonstrated that α-syn plays an active role in different processes occurring at the membrane during membrane fusion, membrane curvature during vesicle formation, docking, pore formation, regulation of neurotransmitter release, and vesicle recycling (Figure 3a,b and Figure 4).

### 3.1. α-Synuclein and Vesicle Docking

Presynaptic terminals contain hundreds to thousands of SV representing a reserve pool. Docking at the presynaptic PM is a crucial step that allows the physical contact of the vesicles with specialised areas of the presynaptic PM called active zones. When the vesicles initially dock, they are not competent for fusion. A vesicle priming step is therefore necessary to achieve a release-ready state upon calcium elevation and next fusion of the vesicles to the PM can take place. The docking is a highly regulated process that requires the interaction of two proteins located on the membrane of SV, vesicle-associated membrane protein 2 (VAMP2) and synaptotagmin and two PM proteins, syntaxin1 and synaptosomal-associated protein 25 (SNAP25) [114]. Although this protein complex is necessary for vesicle docking, α-syn and its interaction with lipids play an import role in this process (Figure 3a).

Interestingly, Man et al. quantified the stabilisation of synaptic-like vesicles docking to the PM by α-syn using total internal reflection fluorescence (TIRF) microscopy [38]. They discovered that, with a constant concentration of synaptic-like vesicles and varying concentrations of α-syn, the number of vesicles docking to the IPM surface increased with increasing levels of α-syn (a mean of 27 synaptic-like vesicles at 10µM of α-syn compared to 11,5 in the absence of α-syn) [38]. In addition, the estimated residence time for docking synaptic-like SUV doubled at 10 µM α-syn compared to the absence of α-syn. Because of the concentration effect, the authors suggest that several α-syn molecules may contribute to the stabilisation of the docking of a single vesicle. They also tested whether these changes would affect the mechanism of stabilisation of the synaptic-like SUV docked to the IPM surface and found that the synaptic-like vesicles docked to the IPM surface are strongly stabilised by α-syn probably also related to an increase in the amount of α-syn bound to IPM-GM than for IPM alone (see Section 2.2). These data indicate that modifications of the IPM composition may affect the mechanism of stabilisation of the docked vesicles by α-syn. In addition, cholesterol, which accounts for 31% of total lipid components of synaptic vesicles membranes [106], is an important regulator of α-syn membrane binding affinity. Indeed, the presence of cholesterol in the lipid bilayer reduces the affinity of the α-syn region 65–97 for synaptic-like vesicles. The in vitro study shows that, as a result, the overall affinity of α-syn for membrane is reduced and exposure of the unbound α-syn region 65–97 to the solvent leads to an increase in vesicle–vesicle interaction promoted by α-syn. Thus, cholesterol has a significant effect in vesicles clustering in vitro (Figure 2c) [62].

### 3.2. α-Synuclein and Fusion Pore

The fusion pore is one of the intermediate states during the fusion reaction when the vesicle connects to the PM which allows the release of the vesicle contents to the external medium. The fusion pore has a pronounced membrane curvature and is a highly dynamic structure (Figure 3). After opening, it reverts to the closed state or dilates leading to the full fusion with the PM, so that it can open and close several times before releasing or dilating further. A pore that closes after transient fusion leads to recapture of almost intact vesicles. In contrast, regeneration of the vesicles is needed when vesicles fully fuse with the PM in order to maintain the vesicle pool. The vesicle recycling rate is thus an important event in maintaining the homeostasis of exo- and endocytosis mechanisms. In addition, the size of the pore is also an important parameter that controls the release depending on the nature of vesicle cargo as well as the strength of the stimulation. For instance, neuropeptides contained in large dense-core vesicles require a strong stimulation to be released [115], but small SV regulate the release of neurotransmitter via rapid flickering of the fusion pore [116].

The concept of pore formation for amyloid proteins was described earlier in 1993 in the Alzheimer’s disease field by the description of annular shaped oligomers formed by the amyloid Aβ proteins and tau [117], which profoundly influence cellular homeostasis. The existence of an amyloid pore exerting its toxicity through the formation of ion channel pores disrupting the intracellular Ca^2+^ homeostasis was confirmed for Aβ in living cells most recently in 2017. Bode et al. demonstrated that Aβ oligomers, but not monomers and fibres, form ion channels that are toxic in cells [118]. The proportion of pores accounted only for one-third of the oligomer preparation. Thus, the authors suggested that among the potential mechanisms leading to the preferential channel formation, the importance of lipid composition specifically GM and cholesterols for Aβ insertion into the membrane could be an explanation [119,120,121,122]. This concept of lipid composition of membranes influencing the insertion of protein oligomers into membranes emerged also concerning the role of α-syn in PD, with the discovery by the Lansbury’s group of membrane permeabilisation by a pore-like structure formed by annular shaped oligomers [123]. Indeed, α-syn oligomers penetrate in the membrane bilayer and give rise to an annular oligomeric species similar to a pore that acts as a protein channel. This formation of a ring-like structure has been confirmed using different sizes of α-syn oligomers, and this process has been directly associated with an increase in neuronal permeability [124].

More recently, in vitro studies demonstrate that α-syn participates in the fusion pore formation (Figure 3b) by penetrating into membranes and giving rise to the formation of annular pore-like structures that increase cell permeability and calcium influx [125]. The authors observed that α-syn affected the fusion pore. Upon α-syn overexpression, an accelerated release is observed preventing the pore closure. Conversely, the loss of α-syn has an opposing effect. Thus, there is a direct relationship between the level of expressed α-syn and the pore dilatation [126]. The ability to expand the fusion pore is not specific to α-syn, since the other synuclein isoforms, the β- and γ-synucleins, share this feature. Overall, this study shows that α-syn facilitates the exocytosis of secretory vesicles by increasing the rate of dilation of the fusion pore and the subsequent collapse of the vesicle membrane upon fusion at the active zone of the synapse [125]. This study is also in line with others showing that overexpression increases the rate of peptide discharge [127]; that α-syn has a similar effect on exocytosis of large dense core vesicles in neuronal cells or in PC12 or chromaffin cells [128]. Interestingly, while it was first suggested that α-syn mutations display little effect on exocytosis [128,129], the authors found a selective inhibition of the fusion pore by the mutations A30P and A53T linked to PD, as both mutations failed to accelerate peptide release in these experiments. 

Although the role of α-syn on dilation of fusion pore has been established, some studies show that the formation and expansion of fusion pore are dynamic processes involving changes in membrane curvature, itself regulated by the SNARE protein complex.

### 3.3. α-Synuclein and the Cooperation with SNARE Proteins in Exocytosis

Fusion and exocytosis events require the regulated cooperation of α-syn with other synaptic proteins. In order to achieve the membrane fusion, membranes must overcome energy barriers created by charge repulsion and local dehydration of polar phospholipid head groups and by membrane deformation. The main actors mediating these processes are the SNAREs, main constituents of the SNARE complex to release energy, thereby enabling the bridging of the two membranes in close proximity. This phenomenon leads to the catalysis of membrane fusion (Figure 3a,b).

Several proteins contribute to regulate the SNARE complex, including α-syn. The SNARE complex mediating the fusion of SV with presynaptic PM during neurotransmission is composed of the target-SNAREs (t-SNAREs) Syntaxin-1 and SNAP25, located on the PM and the vesicular-SNARE (v-SNARE) synaptobrevin2/VAMP2 located in the membrane vesicles [130]. α-syn plays a crucial role in stabilising this complex. Burré et al. show that α-syn directly binds to the VAMP2 N-terminal domain through a short sequence in its C-terminal domain (residues 96–100). In support of this, it has been shown that α-syn lacking the VAMP2 protein–binding region (residues 1–95) does not interact with VAMP2 [131]. Similarly, bimolecular fluorescence complementation assays on hippocampal neurons confirm that the α-syn-VAMP2 interaction occurs at the synapse [131]. Moreover, the simultaneous interaction of monomeric α-syn with the acidic membrane lipids induces stabilisation of the tripartite SNARE complex [132]. These studies confirm the crucial role of α-syn in the stabilising the synaptic SNARE complex Syntaxin-1, SNAP25 and VAMP2 at the fusion pore. This evidence supports the role of α-syn as a chaperone of SNARE proteins. This notion is also supported by experiments performed on aggregated forms of non-mutated α-syn, which exhibit an enhanced VAMP2 binding affinity. The consequent increase of the fraction of VAMP2 bound to α-syn and the reduced amount of free VAMP2, reduce the formation of the SNARE complex inhibiting the docking of vesicles to the presynaptic terminal and impairing neurotransmission [133].

Beside the SNAREs other important protein partners are involved in the regulation of fusion event and interact with α-syn (Table 3). Another key regulator of SV trafficking is the Cysteine-String Protein-α (CSPα also known as DNAJC5). The DNAJ domain of the CSPα protein carries out its function by regulating the ATPase activity of the Heat Shock Cognate 70 (Hsc70). CSPα is a presynaptic protein that contributes to the stabilisation of the tripartite SNARE complex in a different way to α-syn. CSPα in complex with Hsc70 and the adaptor protein small glutamine-rich tetratricopeptide repeat-containing protein α (SGTA) acts as SNARE-chaperone, maintaining SNAP25 in the conformational state allowing the formation of SNARE complex [134]. It is interesting to note that genetic variants of the DNAJC family including CSPα/DNAJC5 have been associated with parkinsonism highlighting a functional pathway involved in the disease [135]. Another family of proteins acting at the synapse called synapsins interacts with α-syn and promotes α-syn functions at the synapse [136,137]. Synapsin III plays an important role as a cytosolic regulator of SV mobilisation [136]. In particular, synapsin regulates vesicle motility by influencing the targeting of α-syn to SV. Furthermore, complexin is another synaptic protein involved in the regulation of SNAREs in vivo and in neurotransmitter release through its interaction with SV [138]. The complexin is normally associated with the curved membrane [139] with a high packing defect [140].

Overall, these data demonstrate that α-syn requires the interaction with both lipids and numerous protein partners in order to fulfil its physiological synaptic function (summarised in Table 3). These interactions affect the localisation of α-syn at the synapse and its ability to stabilise the SNARE complex. In different models, the above-mentioned synaptic proteins involved in the SNARE complex formation and regulation could affect the aggregation state of α-syn as well as synaptic events in different manners as reported in Table 3, demonstrating the fundamental role of neuronal α-syn regulation in the pathogenesis of PD. Thus, pathogenic forms of α-syn altering these key interactions may result in altered SV trafficking and neurotransmitter release.

### 3.4. Loss and Overexpression of α-Synuclein in Neurotransmitter Release

Several experiments silencing or overexpressing α-syn levels have been conducted to demonstrate that α-syn acts as a modulator of release of several different neurotransmitters. Mice with α-syn KO show impaired regulation of the synaptic resting pool, but not the readily releasable pool [146]. As postulated by Senior et al. α-syn may be a negative regulator of neurotransmitter release, controlling both the rate of transfer of vesicles to the readily releasable pool and the probability of vesicle fusion to a given synaptic stimulation. In this study, the loss of α-syn in KO mice is suggested to cause an increase in probability of dopamine release from dopaminergic synapses [147]. Triple KO mice deficient in the proteins of the synuclein family (α-, β-, and γ-synucleins) show that synucleins are important factors to determine the synapse size [148]. Guo et al. also demonstrate that α-syn regulates the dopamine transporter named vesicular monoamine transporter 2 (VMAT2). SNCA KO models increase the concentration of VMAT2 molecules per vesicle [149], while overexpression inhibits the VMAT2 activity leading to increased cytosolic dopamine levels [150]. The activity of other neurotransmitter regulators such as the dopamine transporter (DAT) is also affected by α-syn. [151]. Indeed, WT α-syn interacts through the NAC domain (residues 58–107; Figure 1) with a region in the C-terminal (residues 598–620) of DAT [152,153]. Overexpression of α-syn has been suggested to induce an increased trafficking of DAT from the plasma membrane surface to the cytosol, where it has become toxic due to its ability to induce oxidative damage. In contrast, the overexpression of α-syn leads to a decrease of vesicle density and a reduced dopamine release. Such defects would in turn promote motor deficits [154,155]. Two potential mechanisms could explain such results: α-syn overexpression may (i) affects either the exocytosis or endocytosis of the recycling pool, or (ii) decreases the availability of the vesicle pool. Interestingly, the physiological role of α-syn in dopamine release has recently been better understood based on data obtained in mouse models by Somayaji et al. [156]. They demonstrated that α-syn promotes the dopamine release when neurons in the *substantia nigra* undergo action potential bursts separated by short intervals, in the range of few seconds. The authors suggest that the rapid facilitation may be associated with increased docking and fusion of SV to the membrane of active zones during exocytosis. Conversely, they also demonstrated that a longer interval between two consecutive induced bursts, in the range of minutes, is responsible for a depression of dopamine release that is α-syn-dependent. They proposed that this depression is due to synaptic exhaustion (Figure 4). This α-syn induced presynaptic plasticity is independent on calcium, but depends on the type of neuronal activity [156]. Thus, the authors propose that the dopamine release is strongly dependent on pore size and dilatation as well as on the α-syn protein expression level. In contrast, the release of other neurotransmitters, such as glutamate, is not affected by altered α-syn expression [157]. Additional information on deletion or overexpressing patterns of *SNCA* models are presented in Table 4.

### 3.5. Vesicle Recycling

The neurotransmitter release is a rapid and constant process that continuously requires the availability of newly formed SV. Although de novo synthesis of new SV occurs in the cell body, the main process that ensures the availability of SV pool is the vesicles recycling, a process in which the SV, after the exocytosis and the release of their cargo in the extracellular space are recycled by the cells through the fusion with the PM and the endocytosis. As mentioned previously, α-syn overexpression inhibits exocytosis, but the recycling of SV is also altered [168]. Indeed, this negative effect is mainly associated with the ability of dimers of α-syn to cluster SV leading to reduced vesicles mobilisation which blocks vesicle recycling at the PM [169].

Recently, an in vivo study also showed that the α-syn-112 isoform, produced by in-frame excision of exon 5, inhibits SV recycling. This inhibition is associated with the increased affinity of α-syn 112 for phospholipid binding and enhanced tendency to oligomerise. The same inhibitory effect has been found for α-syn-140 and the α-syn mutant A53T, particularly upon increased synaptic stimulation resulting in loss of SV and expansion of the PM [164]. 

### 3.6. Aberrant α-Synuclein in PD-Lipid Binding and Synaptic Function

The maintenance of a physiological and ordered α-syn conformation is among the parameters that influence its lipid-binding properties and functions, as mentioned above. Indeed, it is proposed that the pathological oligomerisation of α-syn and the formation of α-syn protofibrils lead to synaptic dysfunctions and neurotoxicity [170,171]. The conformation and folding of α-syn influence the behaviour and function of α-syn at the synapse. Although mutations of α-syn can affect its folding, lipid binding, and consequentially its function, there is evidence to suggest that α-syn dysfunctions at the synapse may be an early step in pathogenesis of PD [172,173,174,175], but the exact mechanism leading to pathology remains still unknown.

#### 3.6.1. Oligomerisation of Pathogenic α-Synuclein and Lipid Binding

The point mutations associated with PD could promote oligomerisation and/or aggregation of α-syn by inducing alterations in the secondary structure and, thus, affecting lipid binding properties [176]. PD-related missense mutations are mostly located in the N-terminal region that interacts directly with lipid membranes. In vitro studies show that among the different pathogenic *SNCA* mutations (Figure 1), the A30P is a mutant defective in binding to phospholipids in membrane vesicles, while the A53T mutation has no effect on lipid binding [45,177]. Although the majority of α-syn mutations occurs in this membrane-binding site, not all have a reduced affinity for membrane binding, thus the effect of the A30P mutant is probably due to the presence of the proline residue, which is an amino-acid known to favour destabilisation of the α-helix secondary structure formation [45]. In vivo experiments corroborate these data. The A30P mutant reduced the α-syn interaction with membranes in rat isolated vesicles [178]. Furthermore, high frequency stimulation is responsible for depleting the dopamine storage pool. Interestingly, in mice overexpressing human α-syn A30P, a lower decline in dopamine release was observed after repeated stimulations compared to WT control mice. This effect is directly associated with the decrease in dopamine storage pool in A30P α-syn due to the faster exhaustion of dopamine storage pool compared to WT mice (Figure 4) [158]. These effects of A30P mutation could be explained by an alteration in the folding of α-syn protein leading to a closer association of the N- and C-termini in the mutant protein [179]. 

The A30P missense mutation as well as H50Q, G51D, A53E, A53T are also impacted by different intracellular environmental factors of which the physiological concentration of metals could affect the α-syn oligomerisation. In vitro, trivalent metal ions, such as FeCl_3_ or AlCl_3_, affect oligomerisation by increasing the A30P and decreasing the A53T and moderately decreasing α-syn H50Q, G51D, and A53E oligomer fractions compared to α-syn WT. No difference in oligomer formation was identified for the E46K mutant compared to the WT control [180]. In addition, an in vitro study using the membrane system dipalmitoyl-PC-SUV for which α-syn has strong affinity, shows that the lipid-binding of α-syn A30P and G51D is strongly and moderately reduced, respectively [180].

Since overexpression of α-syn through multiplication of its gene locus is a cause of PD, it is also interesting to note that α-syn overexpression through α-syn lentiviral injection induces a more severe phenotype and dopaminergic neuronal death. This overexpression contributes to increase the levels of some specific lipids such as oleic acid and unsaturated fatty acids [63,181]. In addition, the lipid composition favours or reverses the multimerisation of α-syn. In cell models stably expressing human WT α-syn or PD mutated α-syn, long chain polyunsaturated fatty acids (PUFA) promote α-syn multimerisation, while saturated fatty acids decrease α-syn multimers [79]. 

Thus, the α-syn mutations or α-syn multiplications tend to demonstrate the direct connection between lipids and α-syn oligomer seeding. Knowing that the cellular toxicity induced by α-syn oligomers correlates with their ability to disrupt synthetic and cellular membranes [170], this tends to support the notion of a pathological role of α-syn overexpression in PD. Indeed, Fusco et al. using two different types of α-syn oligomers show that the strain of α-syn oligomers, more prone to disrupt the lipid bilayer of synthetic membrane, localises in the luminal surface of artificial vesicles. In contrast, the α-syn oligomers not associated with cytotoxicity localise to the outer surface of the lipid bilayer [170].

However, the complexity of this relationship between oligomers and membranes is underlined by the recent observation that α-syn overexpression in yeast leads to lipid inclusions lacking the typical fibrillar form of α-syn that has since been considered as hallmark of synucleinopathies. Thereby, oligomerisation is not always observed in α-syn positive inclusion in PD brains. Immunostaining for α-syn in PD neurons shows the presence of irregularly shaped and diffuse inclusion structures, called pale bodies containing organelles and vesicles. Pale bodies have been considered as the first stage in the formation of a mature Lewy body [182]. In addition, the recent work on the composition of Lewy bodies brings out a new scenario supporting the hypothesis that PD is much more than a proteinopathy [41]. Indeed, Lewy bodies are mainly composed of damaged mitochondria, cytoskeletal components, phospholipids, sphingolipids, neutral lipids, lipid droplets (LD), cholesteryl esters, and α-syn oligomers [183]. Thus, this evidence leads to the hypothesis that membrane lipids may have a central role in the seeding, fibrillisation and accumulation of α-syn and that α-syn lipid cross-talk may be among the causes of Lewy pathology [181]. The reciprocal effect of α-syn and lipids points to the central role of both molecules in maintaining cellular homeostasis and probably synaptic functions.

In this context, the molecular cross-talk between lipids and α-syn needs to be further investigated in vivo in order to identify the key processes leading to synaptic dysfunctions.

#### 3.6.2. Fine Regulation of α-Synuclein on Synaptic Activity

The lipid-dependent conformation and/or folding of α-syn influence(s) the α-syn behaviour and function at the synapse. Although the interaction of α-syn with the v-SNARE VAMP2 is well characterised, the exact role of α-syn in SNARE-dependent exocytosis at the synapse remains unclear since contrasting results show both positive and negative role of α-syn in SNARE regulation (Table 3 and Table 4).

In favour of a positive role for α-syn in exocytosis, it has been shown that the conformational change from unfolded cytosolic monomer to the folded α-helical multimers renders α-syn capable of promoting the SNARE complex assembly by clustering VAMP2 molecules during SV docking [184]. Furthermore, the α/β/γ synuclein triple KO mouse model exhibits an impaired SNARE-complex assembly and a consequent loss of synaptic activity. This phenotype is reversed after overexpression of α-syn in α/β/γ synuclein KO neurons in an α-syn dose-dependent manner confirming the crucial role of α-syn in stabilising the SNARE complex [132]. Conversely, inhibitory effects of α-syn on exocytosis have also been described. Indeed, overexpression of α-syn inhibits neurotransmitter release by interfering with vesicle priming [128] or SV recycling [129]. Mice lacking α/β/γ synucleins show increased dopamine release associated with a reduced ability of the nerve terminals to store the vesicle pool. A reduced dopamine-content per vesicle was also detected, suggesting an important role of synucleins in dopamine regulation [185]. A study supporting inhibitory effects of α-syn on SNARE-complex assembly did not observe α-syn/VAMP2 interaction in purified synaptic terminals. Furthermore, the authors demonstrate that in vitro, α-syn reduces the level of arachidonic acid, an important regulator of the SNARE complex, thus affecting its formation and stabilisation [165].

**Figure 5:**
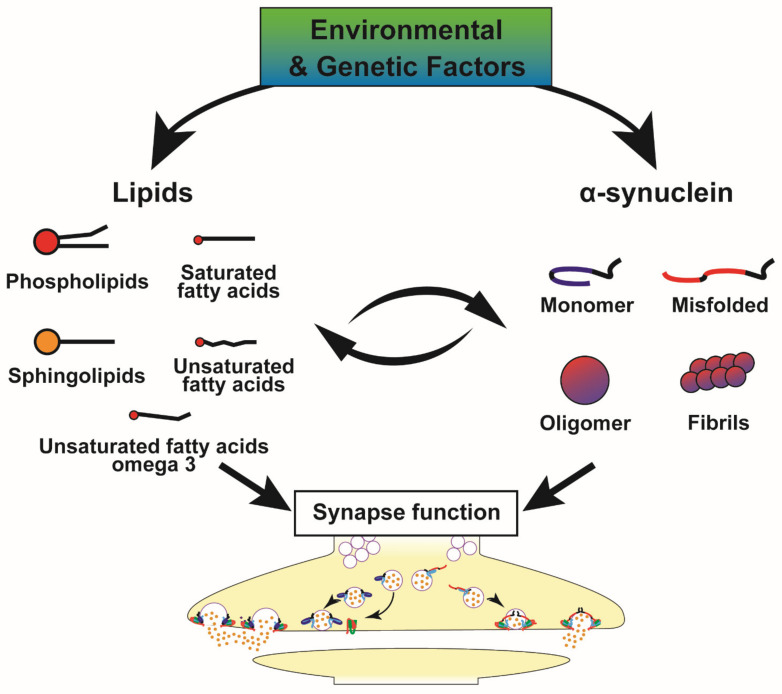
Schematic representation of genetic and environmental factors supporting the role of lipids in PD and synaptic homeostasis. The environmental factors include chemicals and toxins. Among the genetic determinants associated with α-synuclein (α-syn) pathology, many are associated with lipid metabolism or transport, such as *Ataxin2* gene (*ATXN2*), *Chromosome 19 Open Reading Frame 12* gene (*C19orf12*)*, Galactosylceramidase* gene (*GALC*), *Glucosylceramidase* β gene (*GBA*), *Diacylglycerol Kinase Theta* gene (*DGKQ*)*, ELOVL fatty acid elongase* gene (*ELOVL7*), *Phospholipase A2 group VI* gene (*PLA2G6*)*, Scavenger Receptor Class B Member 2* gene (*SCARB2*), *Non-A4 Component Of Amyloid Precursor (SNCA), Sterol Regulatory Element Binding Transcription Factor 1* gene (*SREBF-1*), and *Vacuolar Protein Sorting 13 homolog C* gene (*VPS13C*). Mutations in these genes are responsible for lipid alterations that can trigger the α-syn oligomerisation and consequentially compromise the α-syn synaptic dysfunctions. Aberrant forms of α-syn can also affect the lipids by modifying the membrane integrity [186]. Other parkinsonism-related genes link to vesicular trafficking includes *ATPase H+ Transporting Accessory Protein 2* gene (*ATP6AP2*), *ATPase Cation Transporting 13A2* gene (*ATP13A2*), *Parkinsonism Associated Deglycase* gene (*DJ1*), *DnaJ Heat Shock Protein Family (Hsp40) Member C6* gene (*DNAJC6*), *Leucine Rich Repeat Kinase 2* gene (*LRRK2*), *PTEN Induced Kinase 1* gene (*PINK*1), *Ras-Related Protein Rab-29* gene (*RAB29*), *Ras-Related Protein Rab-39B* gene (*RAB39B*), *Synaptojanin 1* gene (*SYNJ1*), *Synaptotagmin 11* gene (*SYT11*), and *VPS35 Retromer Complex Component gene (VPS35*) may be involved in deregulation of lipid homeostasis (see for review [157]). Physiological synaptic activity mediated by α-syn requires the co-operation of membranes and soluble interactors including lipidic components and protein partners (Table 3). Any disruption of the expression, localisation, interaction of α-syn and/or the above-mentioned partners can induce alterations at different levels of vesicle trafficking processes resulting in an altered neurotransmission and synaptic communication. Membrane phospholipids play an important role in this respect. Indeed, the α-syn-lipid interaction may represent an important step leading to conformational change and physiological multimerisation of α-syn [187]. It seems likely that any variation in membrane lipid composition or expression level of α-syn as well as the presence of α-syn mutant may compromise the α-syn binding properties and functions of α-syn.

Altogether, these divergent studies support the hypothesis that many factors and competitive interactions could regulate the state, the folding and the conformation of α-syn and thus its activity (Figure 3, Figure 5). Indeed, the differential affinity of α-syn regions (Figure 2) for different classes of lipids leads to the hypothesis that any metabolic dysfunction causing alterations in membrane composition, membrane GM content, or membrane lipid raft organisation could strongly affect the α-syn synaptic function and neurotransmission.

## 4. Metabolic Alterations and Genetic Susceptibility Factors in PD, Implications for the α-Syn-Lipid Interplay

In light of the interplay between α-syn and lipids described in the previous section, it is interesting to verify what insight exists into this interplay in PD patients and models. As shown in Table 2, different classes of lipids are indeed deregulated in PD patient samples and PD animal models leading to pathological alterations of α-syn. Furthermore, the interaction of α-syn with lipids is important for α-syn to interact with synaptic protein partners. As a known example, PS has been shown to regulate the α-syn-mediated docking of SV by facilitating the formation of the SNARE complex. The PUFA are a class of lipids actively involved in SV trafficking and their interaction with the N-terminal segment of α-syn increases the α-syn oligomerisation [65]. Thus, alterations in membrane lipid components are widely observed in PD and, as described above, these data confirm their central role in the maintenance of cellular homeostasis.

Several enzymes involved in lipid metabolism also display abnormal activities in biofluids or brain tissues from PD patients or cellular models (Table 2). An increase in sphingomyelinase activity in PD brains has been reported and it has been associated with increased levels of ceramides that activate apoptotic processes. Inhibition of the enzyme sphingosine Kinase (Sphk1), involved in the regulation of sphingolipid homeostasis, correlates with enhanced secretion and propagation of α-syn. The phospholipase D1 enzyme (PLD1), involved in phospholipid hydrolysis is able to prevent α-syn accumulation by activating autophagic flux. Reduced activity and expression level of this enzyme are observed in post-mortem brain of *patients with Lewy body dementia* [87]. Alterations in glycosphingolipid metabolism are also identified in CSF and blood of PD patients as well as modulation of several lysosomal enzyme activities such as increased β-galactosidase and decreased β-hexosaminidase [91], contributing to the deregulation of lipid levels. In addition, some of the lipids deregulated in PD participate in pro-inflammatory processes (sphingolipids and long-chain ceramides) [188] or in anti-inflammatory phenotypes (short-chain ceramides) supporting the evidence that the above-mentioned metabolic alterations contribute to neuroinflammation, a known hallmark of PD [65,189]. Different mechanisms are involved including inflammasome activation, altered calcium homeostasis, changes in the blood–brain barrier permeability and recruitment of peripheral immune cells [91].

Moreover, several studies support a lipid dysfunction in PD that not only affects α-syn, but also actively participates in PD pathogenesis. This new hypothesis is supported by the recent advances in the genetic studies of PD/parkinsonism as well as susceptibility genes associated with α-syn deposition are involved in lipid metabolism as described in the Figure 5 and Table 5, thus shedding light on lipid alterations as important contributors or determinants of synucleinopathies. Moreover, several parkinsonism-related genes including *ATPase H+ Transporting Accessory Protein 2* gene (*ATP6AP2*), *ATPase Cation Transporting 13A2* gene (*ATP13A2*), *Parkinsonism Associated Deglycase* gene (*DJ1*), *DnaJ Heat Shock Protein Family (Hsp40) Member C6* gene (*DNAJC6*), *Leucine Rich Repeat Kinase 2* gene (*LRRK2*), *PTEN Induced Kinase 1* gene (*PINK*1), *Ras-Related Protein Rab-29* gene (*RAB29*), *Ras-Related Protein Rab-39B* gene (*RAB39B*), *Vacuolar Protein Sorting 13 homolog C* gene (*VPS13C*)*,* *VPS35 Retromer Complex Component* gene (*VPS35*), *Synaptojanin 1* gene (*SYNJ1*), *Synaptotagmin 11* gene (*SYT11*) (see for review [157]) are actively involved in membrane and vesicle trafficking and are (or may indirectly be) associated with deregulation of lipid homeostasis supporting this view. 

All of the above-mentioned metabolic and genetic dysfunctions contributing to development of PD or α-syn-related pathologies (Table 2 and Table 5, Figure 5) emphasize the need to further investigate the interplay at the synapse between lipids and α-syn. This is all the more important as several studies tend to show that synaptic dysfunctions occur early in the development of disease [174,175]. Moreover, a reduction in dopamine release as well as alterations of proteins involved in the exocytosis of SV occur prior to the dopaminergic cell death [190]. Given that α-syn is a key determinant of several synucleinopathies, it is thus of great interest to further investigate these altered pathways in multiple system atrophy, dementia with Lewy bodies and Alzheimer’s disease.

## 5. Future Directions

### 5.1. Towards Further Fundamental Advances

Despite these exciting recent progresses, numerous questions remain to be resolved in order to better understand the interplay between α-syn and lipid membranes and their role at the synapse in the different steps leading to neurotransmission. Indeed, vesicle fusion events as well as transient interactions of intra-membranous proteins with cytosolic and cytoskeletal partners make the biological membrane a highly dynamic system, undergoing constant rearrangements during vesicle and membrane trafficking. Thus, artificial systems used in in vitro studies miss the complexity of biological membranes and do not take these parameters into account. Moreover, the physiological state of α-syn is influenced, as mentioned earlier, by various intra- and extra-cellular stimuli including temperature and pH variations, protein interactions, metal ion concentrations and ionic strength. Simplified artificial systems make it difficult to interpret all the combined parameters and hinder the extrapolation of results to in vivo or human models. Thus, future research should develop new tools capable of integrating the complexity of intracellular environment. The conformational change of α-syn, induced by interactions with membranes is transient and occurs rapidly during the physiological synaptic activity. Consequently, the membrane mimetic models should consider the dynamism of α-syn conformations. To date, most of the research has focused on the main α-syn 140 isoforms. However, several α-syn post-translational modifications (acetylation, phosphorylation, glycosylation, ubiquitin conjugation, etc.), as well as several types of α-syn isoforms exist and their relationships with different classes of lipids are still in their infancy [56]. The main α-syn isoforms in the brains are the α-syn 140 AA and the α-syn 112 AA, but there are others, including α-syn 98 or 66 AA, as well as the α-syn fragments including α-syn 1–96 and α-syn 65–140 identified in human brains [212]. Moreover, these α-syn fragments and truncated forms have been identified in other synucleinopathies including multiple system atrophy and dementia with Lewy bodies [213]. In addition, the effect of different types of oligomers in the interaction with membranes in both physiological and pathological conditions has yet to be deciphered. It will thus be interesting to define more precisely the lipid interplay with each of the different types of α-syn forms and post-translational modifications of α-syn. 

In addition, altered lipid levels and metabolic pathways associated with PD and other synucleinopathies evolve with disease progression; in this respect, it is of interest to perform lipidomic analyses at different stages of the disease in order to assess the effect of lipid alterations on α-syn-dependent synaptic activity.

### 5.2. Towards Target Identification and Pharmacological Strategies

Different classes of lipids are actively involved in synaptic functions and, some of them affect the α-syn homeostasis by modulating its conformation, aggregation and finally its cytotoxicity (Table 2). In this regard, future strategies could emerge to modulate the impact of certain lipids in neurodegenerative disorders including synucleinopathies. To date, promising therapeutic approaches aim to modulate the levels of lipids by targeting the activities of proteins involved in the metabolism of the lipid pathway, including enzymes or lipid transporters. In addition, given the neuroprotective effects of some lipids, their direct administration is emerging as a promising strategy to alleviate α-syn cytotoxicity. Other therapeutic strategies point to alleviate conditions associated with PD caused by environmental factors. Finally, synaptic proteins have also been analysed as potential target for therapeutical strategies aiming to restore synaptic function in animal models of PD. Several examples for each of these approaches are detailed below.

#### 5.2.1. Targeting Membrane Lipids or α-Synuclein Membrane Affinity

The α-syn-mediated toxicity in neuronal cellular models could be improved by inhibitors of the stearoyl-CoA desaturase enzyme [80]. The same effect is observed in cellular models overexpressing an engineered α-syn characterised by E35K + E46K + E61K mutations, which leads to the formation of round inclusions [214]. These observations suggest that inhibition of fatty acid desaturation could prevent the oligomerisation and α-syn-mediated toxicity. Based on such evidence, development of strategies to decrease the oligomerisation and aggregation of α-syn might be promising in particular for PD patients, heterozygotes or homozygotes for *SNCA* mutations, or gene multiplication. In this context, decreasing oleic acid production by stearoyl-CoA desaturase (SCD) inhibitors is emerging as potential strategy to rescue α-syn cytotoxicity [58]. An in vitro study also demonstrates a potential protective role of arachidonic acid, which is able to induce the formation of ordered and α-helical structured α-syn oligomers, resistant to fibrillisation [215]. 

Another option is to target lipids participating in synaptic functions, acting on GM which are identified as important factors in maintaining neuronal function [59] and because a consistent decrease in GM brain content has been observed in PD (Table 2). In addition, the intranasal infusion of GD3 and GM1 gangliosides alleviates α-syn toxicity and improves the function of midbrain dopaminergic neurons [216].

Interestingly, a recent approach aims to modulate the affinity of α-syn for membranes and, among them, a promising molecule has emerged known as the anti-microbial squalamine [217].

#### 5.2.2. Environmental Factors and Potential Therapeutic Strategies

Interestingly, α-syn binds to LD, a lipid storage organelle contributing to energy metabolism [218,219]. Overexpression of α-syn in neuronal cells induces accumulation of LD which, in turn, increases the amount of proteinase K-resistant forms of α-syn, suggesting a potential pathological role of LD in PD [220]. Several environmental factors deeply affect lipid homeostasis and, among them, diet plays an important role. Indeed, dietary nutrients are the main substrates of the gut microbiota, which can process and metabolise them. Conversely, dietary nutrients can have an impact on the composition and metabolic activity of the gut microbiota [221]. These processes lead to the productions of intermediate metabolites that profoundly affect host energy homeostasis as well as glucose and lipid metabolism. Studies in animal models confirm that the cross-talk between the gut microbiota and the dietary lipids contributes to the regulation of lipid levels in biofluids and tissues [222]. 

In addition, increased LD formation in dopaminergic neurons [168] has been correlated with iron accumulation, a condition described both in PD patients and in animal models. Iron accumulation is also responsible for lipid peroxidation which, in turn, activates a caspase-independent cell-death pathway known as ferroptosis. Of major interest, pharmacological administration of the iron chelator deferiprone, reduces the abnormally high deposition of iron in the SN, as evidenced by a reduction in the progression of motor deficits in a clinical trial in early-stage PD [223]. The potential of this molecule as a PD modifier is currently being tested in an ongoing phase 3 clinical trial [224].

#### 5.2.3. Targeting Synaptic Proteins

Although many therapeutic strategies aim to target lipids, other approaches directly target synaptic proteins. Among synaptic proteins, CSPα acts as anti-neurodegenerative molecule; this evidence is supported by studies in CSPα-deficient animal models showing impaired synaptic function. Of note, in CSPα-KO mice overexpression of α-syn WT rescues the SNARE-complex assembly deficit and this positive effect is associated with the ability of α-syn to interact with phospholipids. Indeed, the rescue of SNARE complex formation is not observed for A30P, the α-syn mutant with reduced phospholipid binding. These different observations tend to demonstrate the importance of CSPα in the prevention of neurodegeneration. The neuroprotective role of CSPα was reinforced by the demonstration from Spillantini’s group [225] that viral injection of CSPα into transgenic mice expressing a truncated human α-syn (1–120) reduces α-syn aggregates. They first observed that α-syn aggregation at the synapse is associated with a decrease of CSPα, suggesting that α-syn synaptic aggregation affects the CSPα levels. Its function is also affected, as they observed a reduction in the CSPα/Hsc70 complexes with STGa in the striatum. In cellulo, they found that overexpression of CSPα rescues the alteration of vesicle recycling induced by α-syn overexpression. These data confirm that CSPα is an interesting synaptic target. Another target of interest is the synapsin III. The absence of this synaptic protein prevents the formation of α-syn aggregates in primary rodent dopaminergic neurons as well as a reduction in α-syn oligomers and a reduced level of α-syn S129 phosphorylation. Therefore, the loss of synapsin III displays protective effects on synaptic damage and neurodegeneration [144] and also confirms the central role of synapsin III on α-syn aggregation. 

Altogether, these recent experiments provide optimistic perspectives in terms of potential targets for successful therapeutics for synucleinopathies. 

## 6. Conclusions

All of the above-mentioned studies have clearly illustrated that, although the interactions between α-syn and lipids were identified from the first characterisation of the α-syn primary structure, the roles of lipids in the pathophysiology of PD have recently come to light in an insistent manner, like an elephant in the room. This central role of lipids is sustained by many pieces of evidence: (1) lipids and degenerated organelles represent the most abundant components of Lewy bodies [41] (Section 2); (2) α-syn shows a differential binding according to the compositions of inner and outer leaflets of plasma membranes (Section 2); (3) lipid membranes are directly involved in different steps of synaptic exocytosis (Section 3, Table 6); (4) an increasing number of genes associated with α-syn deposition is directly associated with lipid metabolism (Table 5, Section 4); (5) an increasing number of PD-genes affecting α-syn homeostasis is directly or indirectly related to lipid metabolism, such as those acting in vesicular and membrane trafficking (Table 5, Figure 5) [47]; (6) recent advances in therapeutic research show that lipid modulation can directly alleviate the α-syn pathology as well as the synaptic dysfunction (Table 2, Section 5). All these data illustrate the interplay between α-syn and lipids, and suggest that, at least under certain conditions, lipids could contribute to the development of the disease. For these reasons, it seems clear that lipids may contribute to the synaptic dysfunctions leading to PD, highlighting the need to better characterise the lipid/α-syn relationship in vivo. These exciting recent progresses, also point to numerous questions yet to be resolved in order to better understand the interplay among α-syn, lipid membranes, and their role at the synapse in the different steps leading to neurotransmission (Box 1).

Box 1 **Unsolved issues going forward in the field of the interplay among α-syn, lipids, and their role at the synapse**

What are the mechanisms by which pathological α-syn oligomers physically interact with lipid bilayer and affect cellular homeostasis? Different hypotheses have been proposed: 1) α-syn oligomers form an annular pore-like structure, similar to an ion channel, highly dynamic and capable of switching from open to close conformation and allowing the non-selective passage of ions with a consequent alteration of cellular homeostasis. 2) α-syn oligomers, when bound to membrane phospholipids by electrostatic interactions, cause the thinning of lipid bilayer with consequent membrane leakage. 3) Binding of α-syn oligomers to bilayer packing defects induces the extraction of phospholipids with consequent bilayer instability and degradation 4) α-syn trimers and tetramers induce the formation of a lipoprotein particles, called nanodiscs, which are ring shaped by their ability to wrap around the phospholipid bilayer [43].

Are the interactions between α-syn-oligomers and membranes identified in reconstituted systems translatable in vivo? Although the mimetic membrane systems have helped to define the affinity of single α-syn domain for specific classes of lipids (Figure 2), they do not consider the complexity of the biological system. There is therefore a need to further validate these interactions in in vivo models with more sensitive tools, and in the future, to integrate into these analyses several factors such as α-syn oligomers heterogeneity, size, intracellular amounts, kinetic transitions, post-translational modifications, and parameters membranes-related, such as phospholipid bilayer asymmetry and compositional change.

What is the effect of pathogenic α-syn oligomers on membrane homeostasis according to the stage of disease progression? Indeed, the α-syn oligomerisation, the metabolic alterations, as well as the variation in lipid content in brain and biological fluids, change over the time. Moreover, based on the central and direct role that phospholipids have in synaptic functions, it will be necessary to estimate the cytotoxic effect in vivo of α-syn oligomers on phospholipid bilayers. This will help to better elucidate the correlation between structural membrane alteration and PD pathophysiology and related disorders.

## Figures and Tables

**Table 1:** Presentation of membrane models used to investigate the basic physical and biochemical role of α-synuclein (α-syn). The artificial membrane systems are used to study the physical interaction of α-syn and lipids. They are classified into two categories according to their three-dimensional organisation: vesicular and planar models. These systems can be created using different types and proportions of phospholipids allowing the study of different binding properties of α-syn.

Membrane Model	Description	Principal Fields of Investigation
**Vesicular systems**		
**Micelles**	Spherical and monolayer system of amphipathic molecules. Substantial difference with biological membranes.	To identify the conformational change of α-syn domains upon interaction with lipids [42].
**Liposomes**	Spherical vesicles composed of at least one lipid bilayer and of different sizes and curvatures [43] (1) SUV* of 10–100 nm; (2) LUV* 100 nm; (3) GUV* 1 μm.	To investigate the effect of membrane curvature on α-syn oligomer–membrane interactions based on the size: (1) SUV interaction of α-syn with SV; (2) LUV mimicking cell membrane organelles; (3) GUV α-syn relationship with cell membrane [43].
**Planar systems**		
**Lipid monolayer or bilayer**	Planar structure composed of one or two layers.	To investigate the interaction between oligomers and membranes and to analyse the effect of α-syn oligomers on membrane disruption [43].
**Nanodisc**	Planar bilayer structure composed of (1) phospholipids of artificial or cell membrane origin. (2) scaffolding proteins or polymers conferring stability to the system. Size variability from 7 to 50 nm. High similarity to biological membranes.	To allow structuring of disordered proteins, such as α-syn into non-toxic α-helical structures [44].

Legend. α-syn = α-synuclein, GUV* = giant unilamellar vesicles, LUV* = large unilamellar vesicles, SUV* = small unilamellar vesicles, SV = synaptic vesicles.

**Table 2:** Overview of the main lipid classes altered in PD patients and models and their effect on α-synuclein (α-syn). This table provides some examples of different classes of lipids (first column) whose levels are altered in samples and biofluids from PD patients compared to controls (second column). We have also reported examples of enzymes associated with lipid metabolism whose activity is deregulated in PD as well as examples of genetic risk factors for PD associated with lipid catabolism. In some cases, these alterations may directly affect the properties and homeostasis of α-syn (third column).

Lipid Classes	Alterations in PD Patients	Effects on α-Syn
**Phospholipids**		
Phosphatidylcholine (PC)	Decreased PC (34:5, 36:5, and 38:5) in the frontal cortex of PD brains [63]. Decreased PC species with polyunsaturated 3, 4, and 36 carbon in visual cortex of PD [63]. Increased PC 44:6 and 44:5 and decreased PC 35:6 in the plasma of PD patients [63]. Deregulated PC pathway across transcriptome data derived from SN and putamen of PD patients versus controls [64]. Increased PC in CSF of PD patients [65].	POPC bilayer affects the α-syn aggregation [66].
Phosphatidylethanolamine (PE)	Reduced PE in early PD but not in advanced PD [67]. Deregulated PE pathway across transcriptome data derived from SN and putamen of PD patients versus controls [64].	Reduced levels of PE in the phosphatidylserine decarboxylase deletion mutant (*psd1*Δ) increase cytoplasmic α-syn inclusion and enhance toxicity in yeast [68].
Phosphatidylinositol (PI)	Decreased PI in rat and human cortical neurons overexpressing α-syn [63]. Deregulated PI pathway across transcriptome data derived from SN and putamen of PD patients versus controls [64].	Decreased PI species in yeast as well as rat or human cortical neurons overexpressing α-syn [63].
Phosphatidylserine (PS)	Increased PS with 36:1, 36:2 and 38:3 fatty acyl side chains in PD frontal cortex [69]. Deregulated PS pathway across transcriptome data derived from SN and putamen of PD patients versus controls [64].	Facilitation of SNARE complex formation and SNARE-dependent vesicles docking upon α-syn interaction with PS and v-SNARE [69]. Accelerated aggregation on POPS bilayers compared to POPC [66].
**Sphingolipids**		
Sphingomyelin	Reduced in PD anterior cingulate cortex compared to controls [70]. Deregulated sphingomyelin pathway across transcriptome data derived from SN and putamen of PD patients versus controls [64].	Increased α-syn transcript and protein levels upon cell treatment with exogenous sphingomyelin [71].
Gangliosides (GM)	Increased in lipid rafts [72]; 22% reduction in GM brain content in PD male patients, with no differences for PD female [60].	Hypothesised to be involved in both inhibition or enhancement of the α-syn aggregation kinetics [73]. Accelerate α-syn aggregation in presence of high GM1 and GM3 ganglioside concentration in exosomes [74].
Ceramides	Reduced total ceramides in PD anterior cingulate cortex compared to controls [70]. Increased in CSF of PD patients [72].	Increased α-syn toxicity as well as α-syn oligomers formation are linked to alteration in ceramide content [75].
**Saturated fatty acids**		
Stearic acid	Increased in lipid rafts [72]. Increased in rat treated with 6-hydroxydopamine (6-OHDA) [76].	Interaction with α-syn [77].
Palmitic Acid (PA)	Increased in lipid rafts [72].	Increased of α-syn expression levels in Thy1-α-syn mouse model after diet enriched in palmitic acid [78].
Palmitoleic Acid	Decreased in CSF of PD patients [72].	
**Unsaturated fatty acids**		
α-linolenic acid	Decreased in CSF of PD patients [72].	Promoted formation of α-syn oligomers and α-syn induced cytotoxicity [79].
Oleic acid (OA)	Decreased in CFS of PD patients [72].	Increased in response to increase concentration of α-syn monomers [63]. Decreased by stearoyl-CoA desaturase (SCD) inhibition reduced α-syn toxicity [80].
**Unsaturated fatty acids Omega-3**		
Eicosapentaenoic acid (EPA)	Decreased EPA in lipid rafts [72].	
Docosanoic acid (DHA)	Decreased DHA in lipid rafts of DLB brain [81]. Increased amount of DHA (22:6) in PD and DLB brains [79].	Increased in α-syn oligomerisation in a DHA dose-dependent manner [79]. Increased accumulation of soluble and insoluble neuronal α-syn in A53T α-syn mice fed with an enriched DHA diet [82].
**Other lipids**		
Lipids with high solubility in aqueous solution and short hydrocarbon chains.	NI	Induced amyloid fibril formation of α-syn [83].
**Enzyme associated to lipid metabolism**		
Sphingomyelinase	Increased activity in PD brain and increased ceramide level [84]. Of note, acid sphingomyelinase (ASMase) encoded by *SMPD1* is responsible for the hydrolysis of sphingomyelin into ceramide and phosphorylcholine and a reduced ASMase enzymatic activity was associated with an earlier age at onset *SMPD1* variants in PD vs. controls. These genetic variants impair the traffic of acid-sphingomyelinase to the lysosomes [85].	Increased α-syn levels in HeLa and BE(2)-M17 dopaminergic cells in *SMPD1* KO and KD [85].
Sphingosine kinase I	Reduced SPHKs activity under oxidative stress evoked by MPP+ [84].	Induced of α-syn secretion and propagation upon SPHK inhibition [86].
Phospholipase D1 enzyme (PLD1)	Reduced activity and expression level of PLD1 observed in DLB post-mortem brains [87].	PLD1 prevents α-syn accumulation by autophagic flux activation [87].
Glucocerebrosidase (GBA)	Reduced GCase activity in the SN and hippocampus of iPD patients [88].	Misfolded GCase interacts with α-syn and induces α-syn accumulation and aggregation [89].
Cathepsins D and E	Increased activity of cathepsin D in *PRKN*-PD-derived fibroblasts [78] or in iPSC-derived dopaminergic neurons from N370S-GBA PD [90]. Increased activity of cathepsin E in blood and CSF from PD patients (See for review [91,92]).	α-syn is degraded by lipid-associated cathepsin D [93].
β-hexosaminidase	Decreased activity in blood and CSF from PD patients [91].	Increased β-hexosaminidase activity rescues the neurodegeneration induced by α-syn in dopaminergic neurons of the rodent SN [94].
β-galactosidase	Increased activity in blood and CSF from PD patients [91,92].	NI

Legend. α-syn = α-synuclein, CSF = cerebrospinal fluid, DLB = Lewy body dementia, GCase = glucocerebrosidase, iPD = idiopathic PD patients, iPSC = induced pluripotent stem cells, MPP = 1-methyl-4-phenylpyridinium, KD = knockdown, KO = knockout, NI = no information, POPC = 1-palmitoyl-2-oleoyl-*sn*-glycero-3-phosphocholine, POPS = 1-palmitoyl-2-oleoyl-sn-glycero-3-phosphoserine, *PRKN* = Parkin gene, SN = Substantia Nigra.

**Table 3:** Synaptic proteins and their relationship to α-synuclein (α-syn) aggregates and vesicular alterations. The first column mentions the synaptic proteins while the second column mentions the models in which these proteins were studied. The third and fourth columns describe positive or negative effects observed on α-syn and vesicular functions, respectively.

Protein	Model	Positive Effect	Negative Effect
**Complexin**	Mice model over-expressing α-syn		Reduction in complexin 2 level in brain extracts from α-syn transgenic mice compared to controls [129].
	Mice α/β-syn double-KO		30% increase in complexins in *α/*β-syn double-KO mice [141].
**CSPα**	CSPα-KO mice		Reduction in SNAP25, Hsc70 and Hsp70 [141]. Impairment in SNARE complex formation [141].
			Increase in SNAP25 ubiquitylation and proteasomal degradation [134]. Reduction in SNAP25 [134,142] and Hsc70 protein levels [134] Impairment in SNARE-complex assembly [142].
	Neurons overexpressing CSPα.	CSPα suppresses the degradation of SNAP25 and Hsc70 and increases their protein levels [134].	
	CSPα-KO mice overexpressing WT or A30P α-syn.	Overexpression of WT α-syn but no A30P rescues the SNARE-complex assembly deficit induced by CSPα-KO [142].	
	Mice expressing a truncated human α-syn (1–120) injected with viral CSPα.	Viral CSPα injection reduces α-syn aggregates [142].	
**SNAP25**	*Snap25^S187A/S187A^* KI mice carrying an unphosphorylated form of SNAP25 (Ser/Ala phospho-dead mutation on position 187).		Increased number of endogenous α-syn aggregates associated with cytoplasmic side of the plasma membrane. Decreased ability of the SNARE complex assembly [143].
**Synapsin III**	AAV-human α-syn injections in synapsin III KO mice.	Reduction in α-syn aggregation [144]. Reduction in the α-syn S129 phosphorylation in synapsin III KO mice in the striatum ipsilateral of an unilateral injection of AAV-human α-syn and no difference in the contralateral striatum [144].	
	Primary rodent dopaminergic neurons synapsin III KO.	Prevention of α-syn aggregation [135].	
	LB-enriched protein extracts from the SN of PD versus control brain samples.		LB-enriched fractions are immunopositive for both synapsin III and α-syn aggregates [145].
**VAMP2**	Rat cortical neurons treated with α-syn aggregates.		Direct binding of VAMP2 with α-syn aggregates. Reduction in VAMP2 and SNAP25 protein level, but no change in Syntaxin1A. 45% decrease in glutamate release [133].

Legend. α-syn = α-synuclein, α/β-syn = α/β-synucleins AAV = adeno-associated viral vector, CSPα = cysteine-string protein-α, Hsp70 = heat shock protein 70, Hsc70 = heat shock cognate 70kDa protein, KI = knock-in, KO = knockout, LB = Lewy body, SNAP25 = synaptosomal-associated protein 25, SNARE = soluble N-ethylmaleimide-sensitive-factor attachment protein receptor WT = wild type, VAMP2 = vesicles associated membrane protein.

**Table 4:** Fine deregulation of α-synuclein (α-syn) in exocytosis. In the table are presented divergent studies describing the role of α-syn as inhibitor or promoter of SV exocytosis. The studies are classified according to the models used and include in cellulo or in vivo α-syn overexpression and α-syn knockout models, as well as in vitro models using recombinant α-syn or artificial membrane vesicles and assays.

α-Synuclein Models	Positive Effect on Exocytosis	Negative Effect on Exocytosis
**α-syn overexpression models**		
PC12 cells and chromaffin cells overexpressing α-syn.		Reduced catecholamine release in both PC12 and chromaffin cells. Accumulation of docked vesicles at the plasma membrane in PC12, but not in chromaffin cells Potential inhibition of the priming of neurosecretory vesicles in chromaffin cells [128].
Transgenic expression of α-syn in CSPα knockout mice.	Rescue the assembly and function of the exocytic SNARE29, preventing neurodegeneration [159].	
Hippocampal neurons overexpressing α-syn.	Enhanced both spontaneous and evoked neurotransmitters release [160].	
Primary rat hippocampal neurons overexpressing α-syn and endogenous α-syn.	Promoted dilation of the fusion pore [125].	
**α-syn KO/deletion models**		
α-syn KO mice obtained by deleting exons 1 and 2 of the *SNCA* gene.	No impairment in structure of synapse, release of neurotransmitters, mobilisation of SV [141].	
α-syn KD by antisense oligonucleotides in hippocampal neurons.		Blocking the potentiation of synaptic transmission
α-syn KO mice obtained by deleting exons 4 and 5 of the *SNCA* gene in embryonal stem cells.		Dramatic loss of reserve vesicles and an increase in synaptic depression [146].
Non-viral gene therapy based on a new indatraline-conjugated antisense oligonucleotide (IND-ASO) to disrupt the α-synuclein mRNA transcription selectively in monoamine neurons of a PD-like mouse model and elderly non-human primates.	Intracerebroventricular and intranasal IND-ASO administration for four weeks in a mouse model with AAV-mediated WT human α-syn overexpression in dopamine neurons prevented the synthesis and accumulation of α-syn in the connected brain regions, improving dopamine neurotransmission [161].	
**α-syn aggregates models and recombinant α-syn treatment**		
Introduction of α-syn aggregates into single dopaminergic neurons via the patch electrode.		Accumulation of α-syn aggregates may chronically activate KATP channels leading to loss of excitability and dopamine release [162].
Synapse treated with recombinant human α-syn-112.		α-syn-112 strongly inhibits SV recycling [163].
Giant Lamprey synapse injected with α-syn.	Accumulation of clathrin coated pits and clathrin coated vesicles [164].	
**In vitro studies**		
Immobilised α-syn on sepharose beads incubated with radioactive arachidonic acid.		α-syn inhibits both exocytosis and SNARE complex formation by decreasing the levels of free arachidonic acid available to the SNARE proteins [165].
Single-vesicle and bulk in vitro lipid-mixing assays with α-syn purified monomer.	The α-syn monomers promote SNARE complex formation [166].	
Single-vesicle and bulk in vitro lipid-mixing assays with α-syn purified oligomers.		Interaction of large α-syn oligomers with VAMP2 Inhibition of SNARE complex formation Inhibition of docking vesicles [166].
In vitro lipid-mixing assay with monomers and oligomers.		Both α-syn monomers and α-syn oligomers induce the clustering of SV. The α-syn mutant T44P/A89P with reduced lipid-binding affinity reduces the clustering of SV by α-syn oligomers in vitro [167].

Legend. α-syn = α-synuclein, AAV = adeno-associated virus, CSPα = cysteine-string protein-α, KATP channel = ATP-sensitive potassium channel, KD = knockdown, KO = knockout, SNARE = soluble N-ethylmaleimide-sensitive factor (NSF) attachment protein receptor, SV = synaptic vesicles, VAMP2 = vesicle associated membrane protein 2, WT = wild type.

**Table 5:** Presentation of some genetic determinants associated with α-synuclein (α-syn) pathology and having a direct relationship with lipid pathways. This table provides some examples of different genes (first column) associated with α-syn pathology and/or to parkinsonism (second column). Mutations in these genes can directly affect the biological metabolism (third column) and, in some cases, the properties of α-syn (fourth column).

Genes	Genetic Determinants Associated with α-Syn Pathologies	Effect on Lipids	Effects on α-Syn
*ATXN2*	Diseases associated with *ATXN2* include Spinocerebellar Ataxia 2 and PD/parkinsonism with LB pathology [191].	Ataxin-2 expansion affects ceramide-sphingomyelin metabolism [192].	NI
*C19orf12*	*C19orf12* is associated with Neurodegeneration with Brain Iron Accumulation disorders with prominent widespread Lewy body pathology [193].	Role in lipid homeostasis [194].	NI
*DGKQ*	*DGKQ* emerged as PD risk factor in independent GWAS studies [195,196] .	Controls the cellular content of diglycerides.	*DGKQ* loss-of-function in PD might potentially leads to enhanced transcription of *SNCA* [197].
*ELOVL7*	*ELOVL7* identified in GWAS studies as PD-associated gene [198].	FA elongase 7 plays a role in synthesis of long-chain saturated fatty acids involved as precursors of membrane lipids and lipid mediators [199].	Defects in very long chain fatty acid synthesis enhance the toxicity of α-syn WT, A53T and E46K toxicity in a yeast model of PD. The effect on α-syn A30P is inappreciable in a yeast model of PD [75].
*GALC*	Mutations in the *GALC* gene are responsible for Krabbe disease, a demyelinating disorder characterised by the presence of neuronal aggregates, in part composed of α-syn [200].	GALC catalyses the hydrolysis of substrates including galactosylceramide (GalC) and galactosylsphingosine. In PD patients, higher levels of galactosylsphingosine were found respect to controls [201].	Galactosylceramidase treatment improves the survival and health of KD mice, prevents the formation of α-syn in spinal neurons [200] galactosylsphingosine accelerates aggregation of α-syn in a dose-dependent manner [202].
*GBA*	PD risk factor confirmed in GWAS studies [203,204].	Involved in glycolipid catabolism.	The decreased GCase activity identified in CSF and blood PD patients and the consequent increase in glucosylceramide level directly correlates with increased α-syn oligomer formation.
*PLA2G6*	*PLA2G6* is causative for PARK14 in patients with autosomal recessive dystonia-parkinsonism [205].	PLA2G6 hydrolyses the sn-2 acyl chain of glycerophospholipids in free fatty acids and lysophospholipids [206].	Pla2g6 KO mouse neurons show early increase in α-syn/phospho α-syn level [207]. Increased expression of α-syn in cell and animal model with PLA2G6 dysfunction [207].
*SCARB2*	*SCARB2* locus identified in GWAS studies as PD-associated gene. This gene encodes LIMP2 [208].	LIMP2 deficiency can lead to a decrease in GCase activity and α-syn degradation [209].	LIMP2 deficiency can lead to a decrease in GCase activity and α-syn degradation [209].
*SREBF1*	*SREBF1* locus identified as PD risk factor in GWAS [208].	*SREBF1* encodes SREBP-1 that regulates synthesis of sterol.	NI
*VPS13C*	*VPS13C* first mutation identified in a form of early-onset parkinsonism; the pathological features were reminiscent of diffuse Lewy body disease [210].	VPS13C is a lipid transport proteins [211].	NI

Legend. α-syn = α-synuclein, ATXN2 = Ataxin2 gene, C19orf12 = Chromosome 19 Open Reading Frame 12 gene, CSF = Cerebrospinal fluid, DGKQ = Diacylglycerol Kinase Theta gene, ELOVL7 = ELOVL Fatty Acid Elongase 7, GALC = Galactosylceramidase gene, GBA = Glucosylceramidase β gene, GWAS = genome-wide association study, KD = knockdown, KO = knockout NI = no information, PLA2G6 = Phospholipase A2 Group VI gene, SCARB2 = Scavenger Receptor Class B Member 2 gene, SREBF1 = Sterol Regulatory Element Binding Transcription Factor 1 gene, VPS13C = Vacuolar Protein Sorting 13 homolog C gene.

**Table 6:** Summary table on α-synuclein (α-syn) lipid interactions and synaptic dysfunctions in different models. The table provides the main information on α-syn wild type and PD-associated modifications (first column) and their effect on membrane lipids interaction (second column) that has been extensively described in Section 2. The mains functional effects of α-syn on the different steps of exocytosis are also described, highlighting the role of α-syn on the regulation of docking vesicles (third column, explained in the Section 3.1), fusion pore (fourth column, described in Section 3.2), exocytosis (fifth column, Section 3.3 and Section 3.4), and vesicle recycling (sixth column, Section 3.5).

Type of α-Syn Modifications	Lipid Effect	Vesicle Trafficking			
		Docking	Fusion Pore	Exocytosis	Recycling
**WT 140**	Increased α-helical multimers formation.	Increased cluster of VAMP2- vesicles and SNARE complex assembly.			
**A30P 140**	Decreased membrane binding. Decreased membrane curvature. Abolition of interaction with lipid-rafts.	Accumulation of docked vesicles at the plasma membrane. Decreased priming of neurosecretory vesicles.	Perturbation of fusion pore formation.	Decreased catecholamine release. No change in synaptic exocytosis.	
**A53T 140**	Increased multimerisation long chain PUFA-mediated. Decreased multimerisation mediated by saturated fatty acids. No change in lipid binding.	Clustering of VAMP2 SV at the active zone.	Perturbation of fusion pore formation.		Perturbation of SV recycling.
**E46K 140**		Clustering of VAMP2 SV at the active zone.			
K O *		Decreased reserve pool.		Increased concentration of VMAT2 molecules per vesicle.	Increased dopamine release.
**Overexpression**	Decreased membrane curvature induction. Increased oleic acid and unsaturated fatty acid.	Decreased reserve vesicles. Decreased vesicle density.	Prevention the fusion pore closure.	Rescue the SNARE-complex assembly deficit in CSPα-KO mice. Decreased level of synapsins and complexins.	Increased cytosolic dopamine levels due to inhibition of VMAT2 activity.
**Overexpression in CSPα-KO mice**		Rescues the SNARE-complex assembly deficit.			Prevents neurodegeneration.
**WT 112**	Increased phospholipid binding Increased tendency to oligomerisation.				Perturbation SV recycling.
**Soluble aggregates**	Increased aggregates by PUFA.				
**Pathological aggregates**	Increased aggregation by cholesterol, lipids with short saturated acyl chain, GM1, and GM3.	Perturbation of vesicles docking at the presynaptic terminal.		Increased VAMP2 binding affinity.	Alteration in neurotransmission.

Legend. α-syn = α-synuclein, CSPα = cysteine-string protein-α, GM = gangliosides, KO = knockout, PUFA = polyunsaturated fatty acids, SNARE = soluble N-ethylmaleimide-sensitive factor (NSF) attachment protein receptor, SV = synaptic vesicles, VAMP2 = vesicle associated membrane protein 2, VMAT2 = vesicular monoamine transporter 2, WT = wild type. * see Table 4. The effect on exocytosis depends on the knockout model.

## Data Availability

No new data were created or analysed in this study. Data sharing is not applicable to this article.
